# Combining Conventional Organic Solvent Extraction, Ultrasound-Assisted Extraction, and Chromatographic Techniques to Obtain Pure Betanin from Beetroot for Clinical Purposes

**DOI:** 10.3390/antiox12101823

**Published:** 2023-10-02

**Authors:** Davi Vieira Teixeira Da Silva, Diego dos Santos Baião, Alviclér Magalhães, Nathan Farias Almeida, Carlos Adam Conte, Vania Margaret Flosi Paschoalin

**Affiliations:** Instituto de Química, Programa de Pós-Graduação em Ciência de Alimentos e Programa de Pós-Graduação em Química, Universidade Federal do Rio de Janeiro, Av. Athos da Silveira Ramos, Cidade Universitária, Rio de Janeiro 21941-909, RJ, Brazil; davivieira@ufrj.br (D.V.T.D.S.); diegobaiao20@ufrj.br (D.d.S.B.); alvicler@iq.ufrj.br (A.M.); nathan.almeida@gradu.iq.ufrj.br (N.F.A.); conte@iq.ufrj.br (C.A.C.J.)

**Keywords:** betalains, betanin, conventional extraction, ultrasound-assisted extraction, semi-preparative RP-HPLC, ^1^H NMR, LC-ESI(+)MS and betanin antioxidant potential

## Abstract

Red beetroot extract (E162) is a natural colorant that owes its color to betanin, its major red pigment. Betanin displays remarkable antioxidant, anti-inflammatory, and chemoprotective properties mediated by its structure and influence on gene expression. However, the betanin employed in most preclinical assays is a beetroot extract diluted in dextrin, not pure betanin, as no isolated compound is commercially available. This makes its use inaccurate concerning product content estimates and biological effect assessments. Herein, a combination of conventional extraction under orbital shaking and ultrasound-assisted extraction (UAE) to purify betanin by semi-preparative HPLC was performed. The employed methodology extracts betalains at over a 90% yield, achieving 1.74 ± 0.01 mg of pure betanin/g beetroot, a 41% yield from beetroot contents increasing to 50 %, considering the betalains pool. The purified betanin exhibited an 85% purity degree against 32 or 72% of a commercial standard evaluated by LC-MS or HPLC methods, respectively. The identity of purified betanin was confirmed by UV-Vis, LC-MS, and ^1^H NMR. The combination of a conventional extraction, UAE, and semi-preparative HPLC allowed for betanin purification with a high yield, superior purity, and almost three times more antioxidant power compared to commercial betanin, being, therefore, more suitable for clinical purposes.

## 1. Introduction

**Figure 1:**
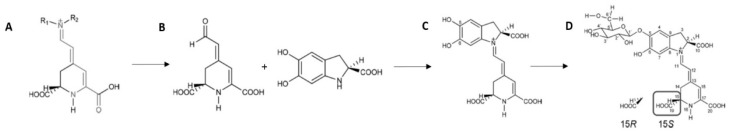
Betalain structure core and betacyanins synthesis (**A**) where betalamic acid condenses with cyclo-3,4-dihydroxyphenylalanine (cyclo-DOPA) (**B**) generating betanidin, the basic structure of the red pigments betacyanins (**C**); betanin is formed by glycosylation of the hydroxyl group of betanidin at C-5. Betanin also exists as its isomer, isobetanin, due to the chiral C-atom of the dihydropyridine unit (**D**); R1: amine or amino acid groups; R2: usually hydrogen. (Modified according to [3]).

Beetroot (*Beet vulgaris*) is the main source of betalains, a water-soluble nitrogen pigment and heterocyclic compound, which can be subdivided into two classes according to their chemical structure: betacyanins, such as betanin (betanidin 5-O-ß-D-glucoside), prebetanin, isobetanin, and neobetanin, responsible for red-violet coloring, and betaxanthins, responsible for orange-yellow coloring, comprising vulgaxanthin I and II and indicaxanthin. Beetroots contain approximately 5–25% betaxanthin and 75–95% betacyanin, and betanin is the most abundant betacyanin found in this tubercle [1] (Figure 1). Betanin, as the phytochemical found in the highest concentration, comprises about 300 to 600 mg/kg of beet tubercle [2].

The use of betanin, as a natural coloring agent, is approved and authorized to be added in “quantum satis” to food products, such as sausage, dairy products, jams, chewing gum, sauces, soups, and as well as a colorant for cosmetics and pharmaceuticals [4,5]. In addition, purified betanin was found to be stable under refrigeration (4 °C) and considered a natural antioxidant for food preservation by inhibiting the formation of malondialdehyde, a product of lipid oxidation, in refrigerated meats [6].

Betanin is a chemoprotective agent, presenting anti-inflammatory effects, strong free radical scavenger ability, and the ability to up-regulate the antioxidant enzyme’s activities. Besides betanin electron donation, it can upregulate the nuclear erythroid-2-related factor 2—antioxidant response element (Nrf2-ARE) and suppress the nuclear factor kappa B (NF-kB) [7,8,9,10,11]. 

The underlying mechanisms of betanin bioactivities are based on key findings in the literature that have been recently compiled and thoroughly outlined [10]. Regarding the antioxidant power, the hydroxyl group of the phenol moiety and/or the cyclic amine group in the betanin structure are both good hydrogen donors conferring reductive properties to betanin [2,7]. In addition, its positively charged moieties confer affinity to negatively charged regions (–CO_2_^−^) of lipids, favoring interaction and protection of lipid membranes. Betanin can also inhibit LDL peroxidation by binding to the polar head of fatty acids or polar residues apoB-100 [12]. On the role of betanin at the transcriptional level by inducing master regulators of RNA synthesis of cytoprotective, antioxidant, and inflammatory genes, betanin can down-regulate Keap1 expression and/or modify Cys residues, culminating in the dissociation of Nrf2, which, in turn, translocate to the nucleus. In the nucleus, Nrf2 dimerizes with the Maf protein and binds to ARE sequences in the promoter region of genes that encode phase II detoxification and antioxidant enzymes [9]. In the inflammatory pathway, betanin acts on ROS, inhibiting the activation of IKK oxidative stress-induced, avoiding IkB phosphorylation and release of NF-kB to the nucleus and then inhibiting the binding between NF-kB and promoter region of target genes, which would activate the transcription of inflammatory cytokines, chemokines, and adhesion molecules. Lastly, betanin, through its role in the dissociation of Keap1-Nrf2 in the antioxidant pathway, makes Keap1 free for inhibitory binding with IKK in the inflammatory pathway [13]. Through these mechanisms, betanin is thought to exhibit potential protection against metabolic disorders and cardiovascular diseases (for review, see Silva et al. [10]).

Various in vitro and preclinical assays have attributed the positive effects of betanin on mechanisms counteracting the development of diabetes and its complications, cancer, inflammation, atherogenesis, and oxidative stress in several cell lines. However, all the effects input to betanin could be underestimated since the majority of pre-clinical trials, with the exception of the study carried out by Silva et al. [14] in animals, have used betanin marketed by several companies such as Sigma-Aldrich Co., TCI America Co., and AdooQ Bioscience LLC, available as a red beet extract diluted with dextrin (7659-95-2) but marketed as the standard for betanin [9,15,16,17,18,19,20,21,22]. This can introduce serious errors since the betanin marketed is not in its pure form, resulting in betanin content overestimated and the biological effects that cannot be input solely to betanin but to other natural compounds present in red beet extract or dextrin that are found in the commercial betanin. In addition, the use of CAS 7659-95-2 implies an overestimation of betanin contents and reported yield in purification assays [23], resulting in the administration of non-effective doses of betanin in the preclinical trials, as well as its incorrect dose-effectiveness calculation, since the mass of the marketed betanin does not correspond solely to betanin.

Obtaining purified betanin for clinical proposals is a challenge due to poor yield extraction and purification of betanin associated with the co-extraction of undesirable compounds bearing the same polarity following its extraction from beetroot tubercles, to the low yield following the traditional purification protocol, the difficulty to stabilize the solid product due to its high hygroscopicity and consequent aggregation and low chemical stability, culminating in the generation of decarboxylated and dehydrogenated derivative compounds. Besides these, the efficient extraction of such compounds is performed by using organic solvents that, in the end, need to be efficiently removed for safe clinical purposes [24,25,26]. On the other hand, obtaining purified betanin is necessary to have a reliable standard to determine betanin content in foods and also the amount of pure compound to be offered to subjects in order to achieve the health benefits already claimed in the improvement of metabolic performance, modulation of redox imbalance and modulation of mediators of inflammatory response in individuals. 

Betanin and other red beet pigments are usually extracted by conventional techniques such as maceration with magnetic stirring or orbital agitation to enable the diffusion of pigments during the solid-liquid extraction using ethanol or methanol [27,28]. 

However, currently, many unconventional methods, such as microwave-assisted extraction (MAE), ultrasound-assisted extraction (UAE), and pulsed electric field (PEF), have been proposed to improve the extraction of pigments [29]. Aqueous ethanol at 30% and ultrasonication seem to exhibit a high extractability of betalains, resulting in high extraction yields of these pigments [30,31].

Herein, we describe the combination of the conventional aqueous ethanol 30% orbital agitation to the UAE and semi-preparative chromatography to obtain betanin with higher purity than those in commercial products marketed worldwide and high yields compared to those reported in the literature. In addition, the antioxidant power of pure betanin obtained was evaluated.

## 2. Materials and Methods

Beetroots (*Beta vulgaris* L.) were purchased at the local trade in the Rio de Janeiro municipality, Southeastern Brazil (22°54′ S 43°10′ W). Reagents of HPLC grade, such as ethanol, methanol, water, and formic acid, were purchased from Tedia Company (RJ, BRA). Betanin (CAS 7659-95-2) was purchased from Sigma-Aldrich Chemical Co. (St. Louis, MO, USA). 

### 2.1. Betalains Extraction through Conventional and Ultrasonic Methods

Beetroot tubercles (150 g) were cut into small pieces, frozen at −80 °C, placed in a borosilicate glass flask, and lyophilized in a Lyophilizer L101 (Liobras, SP, BRA) for 48 h until obtaining a dry powder. 

Betalains were extracted from whole beetroot powder according to Kusznierewicz et al. [32] with some modifications, using aqueous ethanol (30% *v*/*v*) acidified to pH 5.0 with 1% formic acid (*v*/*v*) since betalains have optimal stability at the pH range of 4−6 [29]. Two grams of samples were mixed with 50 mL of extraction solvent and maintained in an orbital shaking incubator G24 Spares (New Brunswick Scientific™, Princeton, NJ, USA) for 30 min at 30 °C. After, the extraction was continued in the ultrasonic bath Vibra-Cell VCX 750 (Sonics & Materials, Newtown, CT, USA) at 165 W and a frequency of 25 kHz for 30 min at 30 °C. The mixture was centrifuged for 15 min at 30,000× *g* and 4 °C, and the supernatant containing betalains was collected. The centrifugation step was repeated 3 times, and the combined ethanolic supernatants were concentrated by a Rotavapor^®^ R-215 (Buchi, Valinhos, SP, Brazil) until dry. Finally, the dried extract was resuspended in deionized water for further analysis.

### 2.2. Betanin Purification by Semi-Preparative Chromatography

The HPLC apparatus consisted of an LC-20A Prominence (Shimadzu^®^, Kyoto, JPN) equipped with a quaternary pump and a DAD model SPD-M20A (Shimadzu^®^). A 15 µm Phenomenex C18 column 250 × 21.2 mm I.D. (Torrance, CA, USA) connected to a fraction collector FRC-10A (Shimadzu^®^, Kyoto, Japan). Elution was performed with a mixture of solvents according to Silva al. [6] with small modifications: solvent A was 1% formic acid, and solvent B, 80% methanol at a linear gradient (0–25 min, 11–55% of solvent B). The sample injection volume was 400 µL at 3.5 mL/min flow rate, and separations were monitored at 536 nm. Red-violet-colored fractions containing betanin were concentrated by rotary evaporation, and pure and concentrated betanin was suspended in deionized water, freeze-dried, and stored at −80 °C for further analysis (Figure S1). 

### 2.3. Photometric Quantification and Comparative UV–Vis Spectra of Beet Samples

Betanin samples were diluted with deionized water at absorption values <1.0 at each respective analytical wavelength. UV–Vis spectra were acquired from purified betanin, betalains pool, and commercial betanin at the range of 300–800 nm using Shimadzu UV 1800 spectrophotometer (Shimadzu^®^) for the quantification of betanin and detection/evaluation of additional bands to evaluate the presence of other compounds than betanin. Betalain contents were evaluated by applying Equation (1), already proposed, for dry-weight samples. The molecular weight (Mw) and molar extinction coefficients (ε) were as follows: Mw = 550 g·mol^−1^ and ε = 60,000 L·mol^−1^cm^−1^, for betacyanin; and Mw = 308 g·mol^−1^ and ε = 48,000 L·mol^−1^ cm^−1^, for betaxanthin [33].

(1) $$BC\;[mg\cdot g^{-1}\;dry\;weight]=\frac{(A\times DF\times Mw\times V)\;}{\varepsilon \times L\times D\times W_{d}}$$

where A comprises absorptions at 536 nm and 470 nm for betacyanin or betaxanthin, respectively, DF is the dilution factor, V is the total volume, L is the cuvette path length, and W_d_ is the dry weight of the red beet material.

### 2.4. RP-HPLC Analysis and Betanin Identification by LC-ESI (+) MS

Purified betanin, betalains extract, and commercial betanin in dextrin were analyzed using a Nucleosil 100-C18 column (250 × 4.6 mm I.D., 5 µm) at a flow rate of 1.0 mL.min^−1^ and 30 µL injection volume. The mobile phase and gradient conditions were the same as those used at the purification step (Section 2.3). Separations were monitored at 536 nm and also at 470 and 280 nm to confirm the absence of other compounds, such as polyphenols and betaxanthins. The purity of betanin and the commercial betanin standard was evaluated by the percentage of the betanin and your enantiomer peak areas in relation to the sum of the area of all other peaks in the chromatogram at their respective λ_max_ and, in the same way, by the intensity of the *m*/*z* signal in the mass spectra of betanin and isobetanin in relation to the peaks generated by the other compounds. Liquid chromatography positive ion electrospray ionization tandem mass spectrometry (LC-ESI(+)-MS) was used to identify purified betanin, according to Silva et al. [6]. The purified fraction was ionized in the positive mode, and ions were monitored in the full scan mode (range of *m*/*z* 150–800). Betanin identification was based on its molecular masses (550 g∙mol^−1^) and by similarity with the literature-available chromatograms and mass spectra [31,34].

### 2.5. NMR Spectroscopy

The ^1^H NMR spectra were acquired using an NMR spectrometer 9.4T Avance III HD (Bruker^®^, Billerica, MA, USA) operating at 500 MHz, equipped with PABBO (BBF-H-D) 5 mm probe head. NMR samples of the commercial betanin (10 mg/mL) and purified betanin (4 mg/mL) were measured in D_2_O at 25 °C. Chemical shifts were reported in ppm using the lock on deuterium oxide as standard. The sample of 1.2:3.5-di-O-isopropylidene-D-xylofuranose (DX, Sigma-Aldrich Co.), used as ERETIC standard, was prepared in 1 mL of Aceton-d_6_ (Cambridge Isotope Lab., Tewksbury, MA, USA) and 19.3 mg of DX, ^1^H NMR spectra of this sample were carried out at 25 °C using standard 90° pulse sequence and 30 s for recovery time delay.

### 2.6. Total Antioxidant Potential of Purified and Commercial Betanin

The purified and commercial betanin were analyzed as previously described by Silva et al. [35]. Aliquots of 900 µL (16 mg·mL^−1^) were diluted in water to achieve the same absorbance, transferred to amber vials, and incubated at 37 °C for 10 min with a solution containing 1 mM Fe^2+^, 10 mM H_2_O_2,_ and 1 mM terephthalic acid (TPA) in 50 mM phosphate buffer (pH 7.4). The hydroxyterephthalic acid (HTPA) mimics the OH· radical, was detected employing an HPLC system equipped with a reverse-phase C18 Kromasil^®^ (Göteborg, Sweden) column (5 µm, 250 × 4.6 mm ID, Kromasil^®^) and RF-10AXL fluorescence detector (Shimadzu^®^) with excitation and emission wavelengths set at 312 nm and 428 nm, respectively. The mobile phase comprised 100 mM sodium phosphate buffer (pH 6.6) at 1.0 mL·min^−1^. The TAP value was estimated by the percentage difference of the HTPA generated by the Fenton reaction, according to Equation (2):

TAP [%] = [((S0HTPA − SHTPA)/S0HTPA)] × 100(2)

SHTPA: surface area of the chromatogram in the Fenton reaction with the sample;

S0HTPA: surface area of the chromatogram in the Fenton reaction without sample.

## 3. Results

### 3.1. Betalains Content and Efficiency of Conventional and Ultrasound-Assisted Extraction Methods

The betalains contents found in beet powder and after the extraction and purification processes are shown in Table 1. The combination of 30% ethanol under orbital shaking and UAE allowed for over 90% betalains extraction efficiency from beet powder, where the betaxanthins extractability percentage was higher than that of betacyanins, 92.8% vs. 82%, respectively. Furthermore, an extracted betacyanin content of 3.50 ± 0.09 mg/g from beet powder was achieved by employing the combined methods. Following the combined extraction, the semi-preparative RP-HPLC technique allowed for the purification of 1.74 ± 0.01 mg of betanin/g of dry weight (DW) from beet powder, comprising a 41% betacyanin content yield from the whole lyophilized beet sample. The amount of purified betanin, as well as beet powder yield, was superior to previously reported results. For example, in one study, 0.34 mg/g of fresh weight (FW) betanin from Algerian red beetroot was recovered by simple solid-liquid extraction in ethanol/water at a 20/80 (*v*/*v*%) ratio. Betanin content recovered was also superior to those achieved by testing different ethanol/water ratios, such as 80/20, 60/40, and 40/60 (*v*/*v*%) [36] following quantification by spectrophotometry but not in its purified form. In another study, reverse phase chromatography (RPC) was applied to purify 0.30 mg/g of a betanin/isobetanin mixture from fresh beetroot, achieving 0.19 mg/g betanin from freeze-dried beetroot samples following Sephadex LH-20 gel permeation chromatography. In the same study, the semi-preparative RP-HPLC method recovered only 0.13 mg/g of betanin/isobetanin from fresh beetroot and 0.12 mg/g from freeze-dried beetroot samples. These purification methodologies were not preceded by extraction steps, which may explain the low recovered pigment contents [37].

In another extraction model, the two-step microwave-assisted extraction (MAE) using a mixture of ethanol and water (1:1) containing 40 mM ascorbic acid resulted in the extraction of 1.87 mg/g of betacyanins from freeze-dried red beet. Although a high total betacyanin content was extracted, the amount is still lower than the one obtained in the present study (3.50 ± 0.09 mg/g), and the betanin fraction was not quantified [28]. 

Ultrasound-assisted extraction using 30% aqueous ethanol for 30 min at 30 °C was found to be the best condition for extracting betalains from red beetroot powder, beetroot juice, beetroot waste powder, and air-dried beet waste when compared to 100%, 50%, and 20% (*v*/*v*) aqueous ethanol or methanol, achieving total betalain extraction ranging from 0 to 3.06 mg/g [31]. Another report indicated a higher betacyanin content of 4.24 mg/g extracted from beet samples employing a circulation oven and optimized conditions of 90 min assisted-ultrasonication at 52 °C in 25% aqueous ethanol as the organic solvent [38]. Aqueous ethanol concentration above 20%, in which polarity is reduced with increasing ethanol concentration, reduces betacyanin extractability, as these pigments exhibit a greater affinity for hydrophilic environments [39]. This may explain the higher total betacyanin content recovered from beet matrices in the aforementioned experiments compared to the present study, where 30% ethanol was employed. A yield of 5 mg/g of betanin from fresh beetroot was achieved following conventional aqueous methanol 80% (*v*/*v*) extraction combined with normal phase chromatography purification. The purified betanin content reported by the authors was very high, but the assay failed when using a commercial betanin (red beet extract diluted with dextrin) as the standard, as betanin contents may have been overestimated [21]. In the present study, the betacyanins contents estimated in the commercial betanin sample were higher than the purified betanin (Table 1), but no extraction method was applied to the commercial standard. Thus, the amounts estimated by spectrophotometry refer to a set of red pigments and not specifically to the betanin mass as determined in the purified sample. 

**Figure 2:**
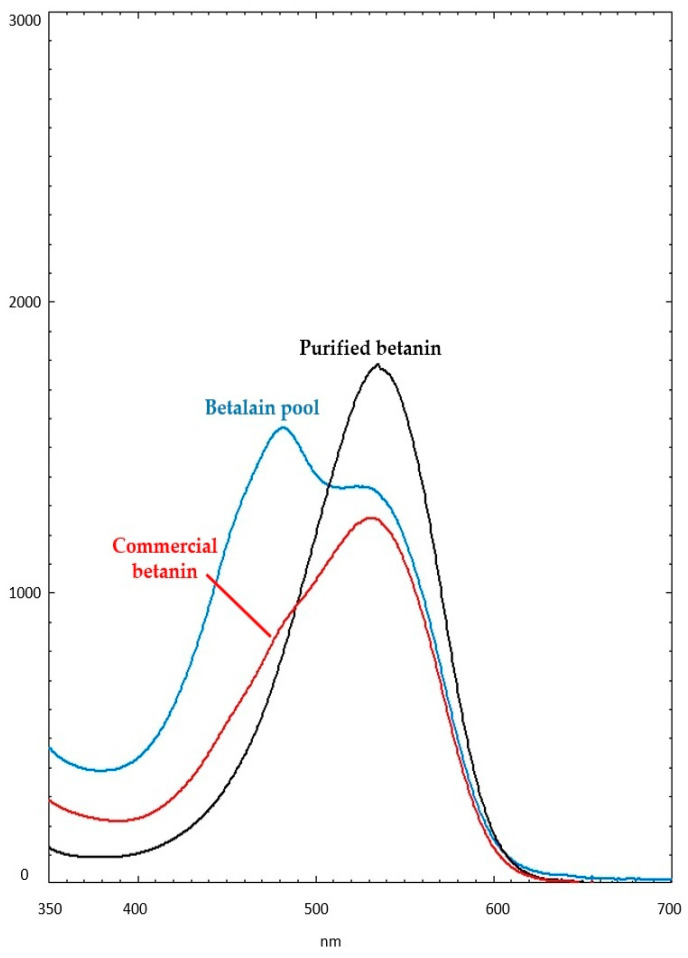
The UV-Vis spectra profiles for purified betanin (black line), betalain pool (blue line), and commercial betanin in dextrin (red line). All compounds were dissolved in deionized H_2_O at a 1:100 (*v*/*v*) ratio.

The UV-Vis spectra of samples in water are depicted in Figure 2. Absorption bands partially overlapped at around 460 nm were observed in the betalain-containing fraction, indicating the presence of betaxanthins at high concentrations, while bands between 500 and 540 nm indicated betacyanins. The most intense band was observed at 460 nm and corroborated with the highest extraction yield (>90%) estimated for betaxanthins in the betalains extract (Table 1). Commercial betanin in dextrin exhibits a broadening of bands between 440 and 480 nm that were attributed to betaxanthins, still present in the sample, and exhibited a more intense band at approximately 530 nm referring to betanin and red-violet pigments (Figure 2). 

A strong maximum absorption band was observed at around 530 nm without band widening for the purified betanin obtained by semi-preparative RP-HPLC following the novel method proposed herein by associating the conventional ethanol extraction to UAE (Figure 2). 

### 3.2. RP-HPLC-DAD Profile of Beet-Derived Pigments and ESI(+)-MS of Purified Betanin

**Figure 3:**
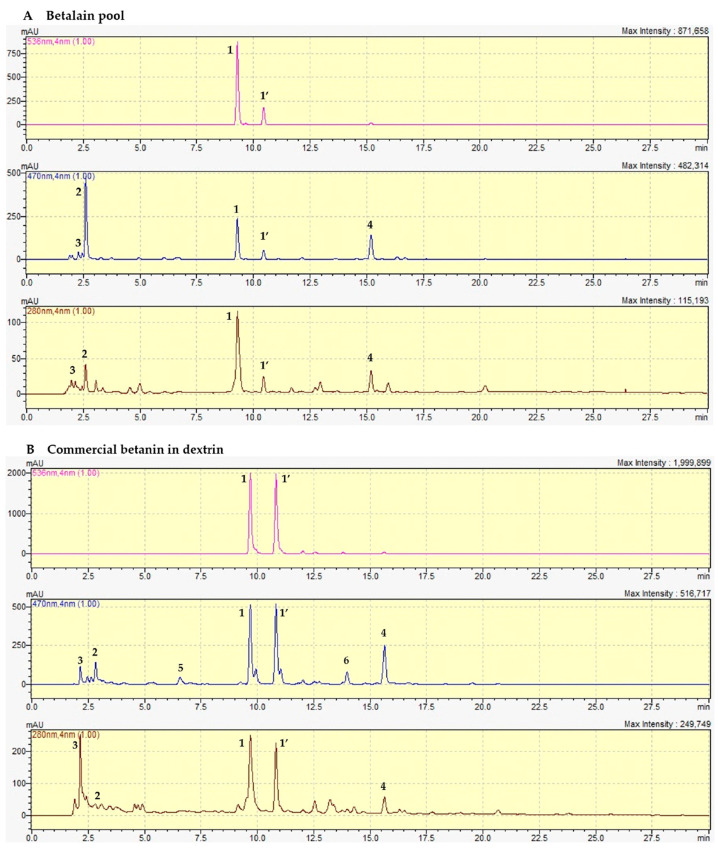

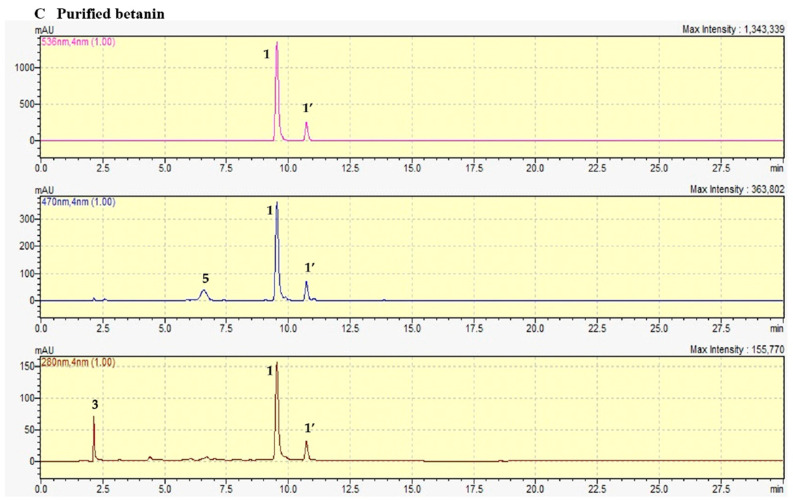
Comparative RP-HPLC-DAD chromatograms of major betalains detected at 536 nm (pink peaks), 470 nm (blue peaks), and 280 nm (brown peaks) from betalains pool (**A**), commercial betanin in dextrin (**B**) and purified betanin (**C**) **1**—betanin, **1’**—isobetanin, **2**—vulgaxanthin, **3**—sucrose, **4**—neobetanin, **5**—2,15-bidecarboxy-xanbetanin, **6**—17-decarboxy-betanin. Chromatographic conditions: mobile phase A—water/formic acid (99/1 *v*/*v*%), mobile phase B—methanol/water (80/20 *v*/*v*%), flow rate at 3.5 mL/min.

The representative chromatograms of commercial betanin, betalain pool, and betanin samples monitored at 280, 470, and 536 nm are depicted in Figure 3. Betanin and other betacyanins exhibit maximum light absorption between 532 and 540 nm, depending on the solvent [40,41]. However, monitoring compound elution only at this wavelength range is a mistake when the aim is to demonstrate purity, as it makes it impossible to identify other compounds with λ_max_ below the betacyanins range, such as betaxanthins, polyphenols, and sugars. For example, Ahmadi et al. [23] monitored the separation of purified betanin from fresh beet exclusively at 538 nm, omitting the possibility of compounds other than betacyanins in the sample.

When monitored at 470 nm, the betalain pool exhibited an intense peak at 2.7 min, identified as vulgaxanthin I (Figure 3A), corroborating the high yield of this compound during betaxanthin extraction (Table 1 and Figure 2). Commercial betanin exhibited a peak at 280 nm, named as peak 3, identified as sodium (Mw 22.9 g·mol^−1^) and sucrose (Mw 342 g·mol^−1^) adduct through the molecular ion *m*/*z* 365 in LC-MS analysis at approximately 2.5 min (Figure S2), as previously reported [42,43]. Adduct generation between metal ions such as sodium and sugars has been generally related in previous reports since sodium is ubiquitous and readily observed as an adduct with carbohydrates, even when sodium salts are not added to the sample [42,44,45]. In addition, commercial betanin exhibited a peak at 2.7 min identified as vulgaxanthin I (Figure 3B), confirming the lack of purity due to the presence of other betalains as observed in the UV-Vis spectra. A peak at 13 min probably represents the decarboxylated derivative orange-red 17-decarboxy-betanin. Another peak eluted at 6.5 min corresponds to 2,15 or 2,17-bidecarboxy-xanbetanin found in both commercial and purified betanin (Figure 3C), indicating carboxyl group degradation products at positions C-2, C-15, and C-17, as betanin is susceptible to decarboxylation by exposure to high temperatures and/or freeze-drying. Both samples were subjected to adverse conditions to ensure solvent evaporation and conversion to HPLC grade. Indeed, C-2, C-15, and C-17 are more prone to decarboxylation by environmental factors, and it is difficult to entirely avoid their occurrence during sample processing (Figure S2) [24,30,37,46]. The commercial betanin and betalain pool also exhibited minor peaks between 280 nm and 470 nm at 11.8 and 21 min, which may comprise decarboxylated and dehydrogenated derivatives, in addition to a more intense peak at 16 min identified as neobetanin (Figure 3A,B). Neobetanin, although exhibiting a yellow color, is a betacyanin naturally present in beetroot but, at the same time, has often been attributed as the major degradation product from betanin dehydrogenation formed during sample processing [24,47,48,49,50].

The chromatographic profile of purified betanin and isobetanin were both monitored at 280, 470, and 536 nm for unequivocal identification, proven there are no other compounds, except for the aforementioned 17-decarboxy-betanin at 6.5 min elution time and a small amount of adduct formed by sodium and sucrose [42,43], generating a molecular ion peak of 365 *m*/*z* (Figure S3), eluting at 1.5 min at 280 nm, although sucrose λ_max_ is between 260 and 270 nm [51,52].

In the present study, the application of the conventional methodology combined with the ultrasonic-assisted treatment followed by semi-preparative RP-HPLC allowed the extraction and purification of betanin and its enantiomer with a purity degree of 85% calculated by the area of all chromatographic peaks at each respective λ_max_, while the commercial betanin standard (CAS 7659-95-2) exhibited a 72% purity. Interestingly, the degree of purity found for purified betanin determined by the mass spectrum was 86%, while commercial betanin exhibited a purity of only 32%, probably due to the high content of sugar and low content of betanin and isobetanin, since they can be more precisely determined by ESI(+)-MS, which is based on the ionized mass of the compounds and not on the Uv-Vis absorption. Furthermore, the LC-MS method presents high sensitivity of determinations, being effective in quantifying compounds at trace levels in samples [53], resulting in more faithful data than those found by HPLC-DAD for the commercial sample. The presence of degradation products due to decarboxylation and dehydrogenation, as noted previously, can prevent obtaining betanin with a higher degree of purity, although this is not an issue, as purified betanin has been found to be stable for 9 months at −30 °C [6]. Purity determination by HPLC was calculated by the sum of areas of the main peaks at their respective λ_max_, which seems more reliable than considering a single wavelength for all peaks, disregarding the individual λ_max_.

The method employed herein allowed for the recovery of 41% pure betanin from the beet matrix and the recovery of 50% pure betanin from the betalain pool obtained after combining ethanol/orbital shaking extraction and UAE, obtaining 1.74 ± 0.01 mg.g^−1^ of betanin from beetroot (Table 1). Previous studies have also described an effective purification procedure by isolating betanin in its purified form but fail when monitoring the elution only at 538 nm and displaying the *m*/*z* spectra solely from 536 to 560 nm, disregarding compounds with lower λ_max_ and different molecular weights [23]. 

Although a high yield of betanin was obtained in the present study, it has been reported that the time and power of ultrasonication can negatively impact the stability of bioactive compounds [54], and this was not evaluated during the extraction of betanin subjected to UAE for 30 min at 165 W. This may be a limitation of the study since the yield could be influenced by the UAE conditions. On the other hand, a number of studies showed that UAE not only contributes to increasing the extraction of polyphenols but also preserves and increases the biological activity of these natural bioactive compounds compared to other conventional extractions [55]. This finding suggests that the same may also occur for the extraction of betalains. Furthermore, the betanin purification yield was calculated for intact betanin molecules, which exhibit their maximum absorbance at 536 nm by HPLC-DAD and *m*/*z* 551 in mass spectrometry. Possible losses in betanin during the UAE procedure should be considered in the future to evaluate the impact of UAE conditions, such as time, power, and frequency, on the yield and betanin molecule stability.

**Figure 4:**
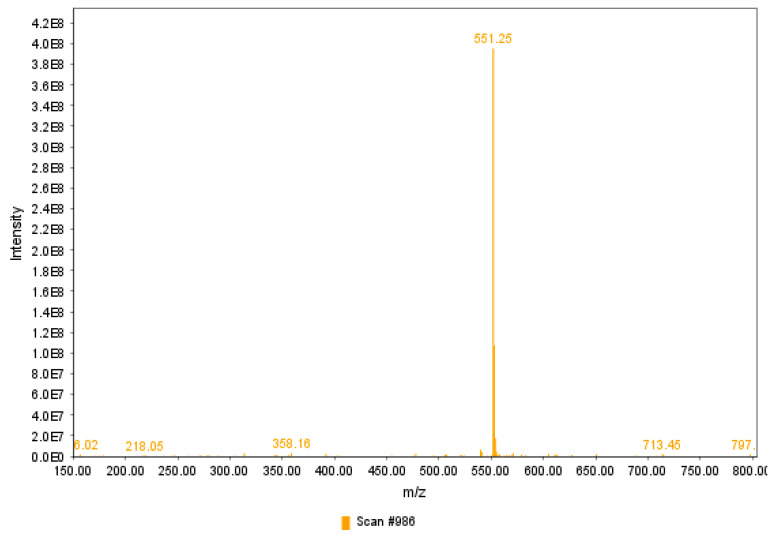
Positive electrospray ionization tandem mass spectrometry ESI(+)-MS of betanin fraction following the semi-preparative purification and mobile phase evaporation, indicating an intense protonated ion at *m*/*z* 551.25 [M+H]^+^.

Figure 4 represents the mass spectra of purified betanin. The positive ionization of purified betanin fraction following semi-preparative RP-HPLC and mobile phase evaporation, evaluated in the range of *m*/*z* 150–800, exhibited the protonated ion *m*/*z* 551 [M+H]^+^, consistent with the molecular mass of betanin of 550 g/mol [6]. Previous analyses of the marketed betanin used as a standard have shown a mass spectrum displaying the ion *m*/*z* 551, but the purity of the standard is questionable, as only a partial scan covering the *m*/*z* 548–549 range was displayed [21].

### 3.3. NMR Spectroscopy

**Figure 5:**
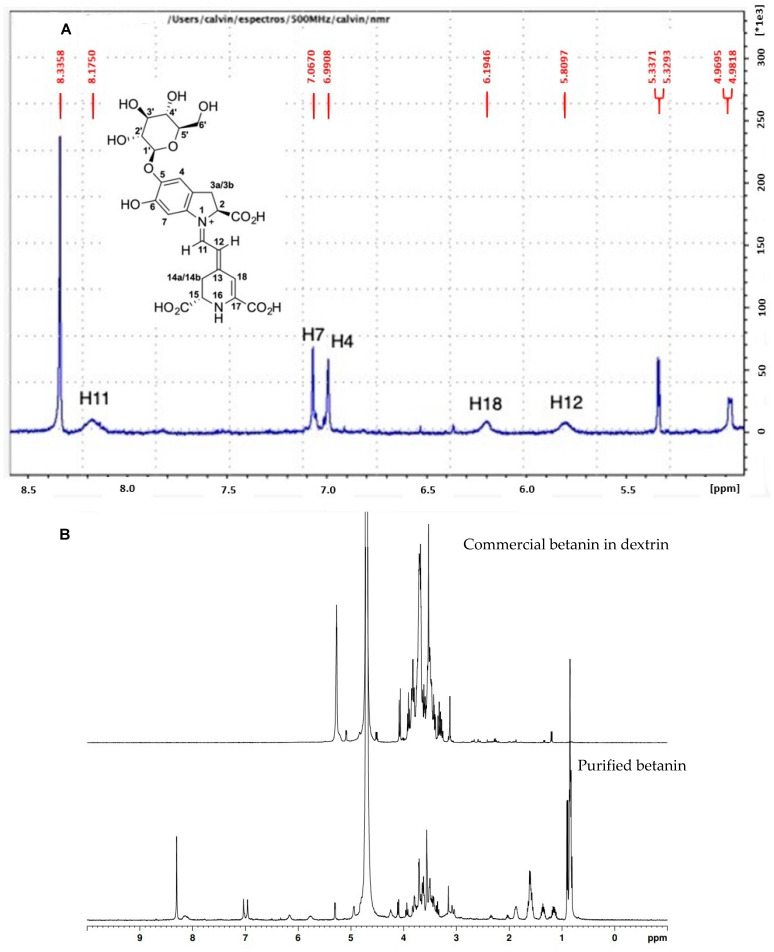
^1^H NMR spectra in D_2_O at 25 °C from purified betanin (**A**) and stacked comparative ^1^H NMR spectra of commercial betanin with purified betanin (**B**).

The ^1^H NMR spectra obtained from pure betanin is similar to betanin’s 1H NMR previously reported [56,57,58,59], where signs can be identified between 6.9 and 7.0 ppm (H4 and H7), attributed to atoms in the aromatic moiety of cyclo-DOPA-5-O-β-D-glucoside. It is also observed the H11 atoms, near 8.2 ppm, and H12 atoms, near 5.8 ppm of the imino group, and the H18 atom, near 6.2 ppm, from the betalamic acid moiety (Figure 5A).

By overlapping the ^1^H NMR spectra of purified betanin to commercial betanin standard, it can be seen that the latter contains the highest concentration of free sugars, which display their characteristic hydrogen signs between 3.0 and 4.3 ppm, approximately, in addition to the anomeric hydrogen of the sucrose molecule at 5.3 ppm (Figure 5B). The high free carbohydrate content found in the commercial betanin spectrum reflects the high sucrose-beet tubercle composition, evidencing a lack of purity of the standard sample. Furthermore, the sugar content found in the commercial sample suppressed the hydrogen signals of the betanin structure, which is present at lower concentrations than sugars (Figure S3). It is important to point out that content of 10 mg/mL of commercial betanin and 4 mg/mL of purified betanin were used as NMR samples, and through the use of 1,2:3,5-Di-O-isopropylidene-D-xylofuranose (DX) as ERETIC standard, it was found 14 times more betanin in the purified sample compared to commercial betanin. This underscores the issue of accuracy when employing commercial betanin in vitro and preclinical studies that ascribe biological effects to the entire mass of the commercial betanin standard, as though it were exclusively comprised of pure betanin. This approach disregards the presence of other compounds, such as sugars and other bioactive substances, which may, for instance, be present in greater quantities than betanin.

### 3.4. Total Antioxidant Potential (TAP) of Purified Betanin and Commercial Betanin

**Figure 6:**
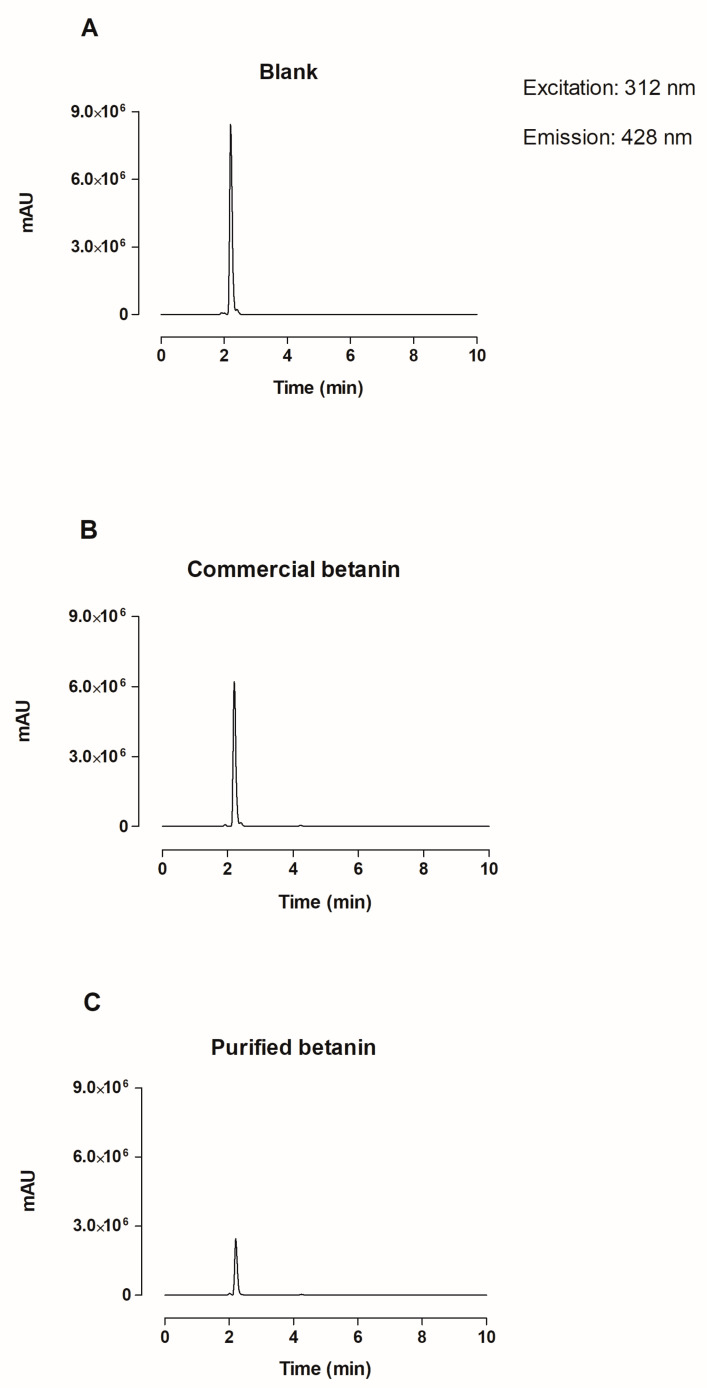
Chromatograms representing the HTPA radical generated by the Fenton reaction at the mixture of 1 mM Fe^2+^, 10 mM H_2_O_2_, and 1 mM TPA in 50 mM phosphate buffer (pH 7.4) during 10 min at 37 °C, without the sample (**A**) and with the addition of 900 µL of commercial (**B**) or purified betanin (**C**), both at the concentration of 16 mg·mL^−1^.

Figure 6 displays the chromatograms of the HTPA radical generated by Fenton reactions before and after adding the samples. Purified betanin exhibited a TAP, i.e., HTPA radical inhibition capacity of 70.91 ± 1.71%, while commercial betanin presented 25.19 ± 1.18%, almost three-fold lower than the purified betanin.

The total antioxidant potential (TAP), defined as the sum of products of concentrations of all antioxidants present in the sample multiplied by their rate constants with the free radicals, was used to evaluate the total antioxidant ability of betanin using the HPLC system. It used a fluorescence detector, which conferred high sensitivity, versatility, and automation without loss of specificity or precision, and showed the antioxidant power of the betanin purified through a decrease in HTPA concentration [60,61].

Although both samples contained the same concentration of 16 mg·mL^−1^, purified betanin resulted in greater OH^•^ radical inhibition, represented by HTPA, as it is composed exclusively of betanin, while the commercial sample is composed of betanin and other pigments, which do not display the same antioxidant power. These findings confirm that the use of purified betanin in biological assays is reliable, as the bioactive compound content applied in the test corresponds only to betanin and not to other betalains naturally present in the commercially available beetroot extract termed betanin. Lastly and most importantly, the betanin obtained in this study, by combined extraction methods followed by chromatographic purification, exhibits the potential to exert biological effects in living organisms since betanin can inhibit highly reactive oxygen species, as evidenced by the Fenton reaction, elicited by H_2_O_2_ and OH^•^ radicals, which can be very harmful to living organisms.

## 4. Conclusions

Betanin was proven adequately extracted through a simple combination of conventional extraction under orbital shaking combined with ultrasound-assisted extraction and semi-preparative chromatography. This combined method may be useful to solve the current gaps concerning the use of commercial betanin for clinical purposes without adequate purity instead of isolated betanin, probably due to the low yield of extraction and purification methods employed. The employment of combined conventional and UAE extractions followed by chromatographic purification makes the use of isolated betanin reliable and can correct errors in non-specific results due to the positive or negative influence of other matrix components from commercial samples, such as other betalains, polyphenols, and high sugar contents. Furthermore, the use of pure betanin avoids erroneous estimation of the dose effect of betanin. The method applied here allowed for an efficient total betalains extraction and high pure betanin yields. Different variables in the combination of the conventional method with UAE, such as time, temperature, power, and frequency of ultrasonication, can be evaluated in the future using box-Behnken design as a tool to improve the method of preparation and, maybe, increase the yield of pure betanin, this being a limitation of the present study. Furthermore, although it was achieved a high purification yield for betanin, the inclusion of stabilizing agents in the future to the isolated betanin, such as cyclodextrin, trehalose, or other polymers, should be considered and tested to determine if they can protect it from degradative reactions caused by environmental factors, such as light and oxygen exposure, or self-decomposition and loss through volatility since all those conditions compromise its stability and its employment as adjuvant to aid in promoting health. Isolated betanin exhibits positive effects on glucose metabolism, antioxidant enzyme activities, and damage recovery of animal hepatocytes following a high-fat diet. In addition, the betanin purified herein is more potent in inhibiting the OH^•^ radical than commercial betanin, as evidenced by the TAP assay. Future animal and clinical trials should be conducted applying the combined extraction and purification method reported herein. Experiments in human cell lineages should be conducted to evaluate the minimal dose effects of pure betanin and elucidate the intestinal mucosal transport of betanin without interference from other matrix compounds. A reliable, reproducible, and high-yield method to prepare pure betanin is required to further study the biological effects of this powerful antioxidant and transcriptional modulator that regulates the cellular redox balance and inflammatory status of living organisms.

## Figures and Tables

**Table 1:** Betacyanins, betaxanthins, and purified betanin contents (mg of each compound/g of freeze-dried red beet) and yields after red beet powder combined extraction.

	Red Beet Powder	Betalain Pool	Purified Betanin	Commercial Betanin
Betacyanins (mg/g)	4.26 ± 0.12	3.50 ± 0.09	1.74 ± 0.01 *	1.79 ± 0.20
Betaxanthins (mg/g)	3.11 ± 0.02	2.89 ± 0.01	-	0.84 ± 0.12
Betacyanins yield (%)	-	86%	41%	-
Betaxanthins yield (%)	-	92.8%	-	-

Content of compounds extracted from red beet powder. Values are expressed as means ± SD of triplicate extractions (*n* = 3), followed by spectrophotometric estimation. * refers to the pure betanin content since it was isolated from the other betacyanins. Red beet powder was obtained from freeze-dried whole beetroot; Betalain pool was obtained by combined extraction; Purified betanin was obtained by combined extraction followed by semi-preparative HPLC; Commercial betanin comprises a red beet extract diluted in dextrin marketed by Sigma-Aldrich Co.

## Data Availability

All methodology data and chromatographic data can be requested from Davi Vieira Teixeira da Silva.

## Supplementary Materials

The following supporting information can be downloaded at: https://www.mdpi.com/article/10.3390/antiox12101823/s1. Figure S1: Purified betanin freeze-dried after betalains extraction employing 30% ethanol under orbital shaking followed by UAE and semi-preparative chromatography; Figure S2: LC-MS spectra of commercial betanin showing sodium (Mw 22.9 g·mol^−1^) and sucrose (Mw 342 g·mol^−1^) adduct through the molecular ion *m*/*z* 365 at approximately 2.5 min; Figure S3: LC-MS spectra of purified betanin showing sodium (Mw 22.9 g·mol^−1^) and sucrose (Mw 342 g·mol^−1^) adduct through the molecular ion *m*/*z* 365 at approximately 2.5 min; Figure S4: Main degradation products of betanin generated after decarboxylation and/or dehydrogenation generated during processing; Figure S5: ^1^H NMR spectrum of commercial betanin at a 24x magnification depicting suppressed H4, H7 and H11 hydrogen signals from betanin molecule due to the high sugar contents.
