# Supplementary material for: Combining Conventional Organic Solvent Extraction, Ultrasound-Assisted Extraction, and Chromatographic Techniques to Obtain Pure Betanin from Beetroot for Clinical Purposes
Source: Antioxidants (Basel). 2023 Oct 2;12(10):1823. doi: 10.3390/antiox12101823 (PMC10604211; doi:10.3390/antiox12101823)
Supplement: Supplementary file 1 [file antioxidants-12-01823-s001.zip › antioxidants-2590751-supplementary.pdf]

Combining conventional organic solvent extraction, ultrasound-assisted extraction, and chromatographic techniques to obtain pure betanin from beetroot for clinical purposes

Supplementary files

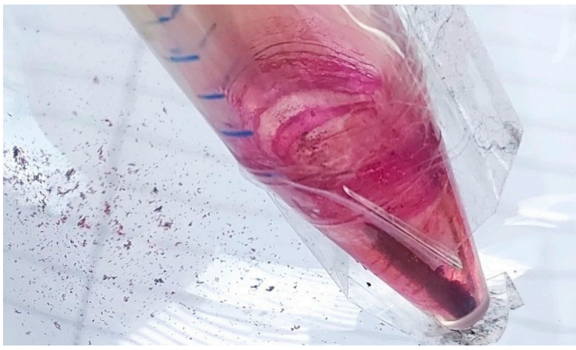

Figure S1: Freeze-dried pure betanin after betalains extraction employing 30% aqueous ethanol under orbital shaking followed by UAE and semi-preparative chromatography.

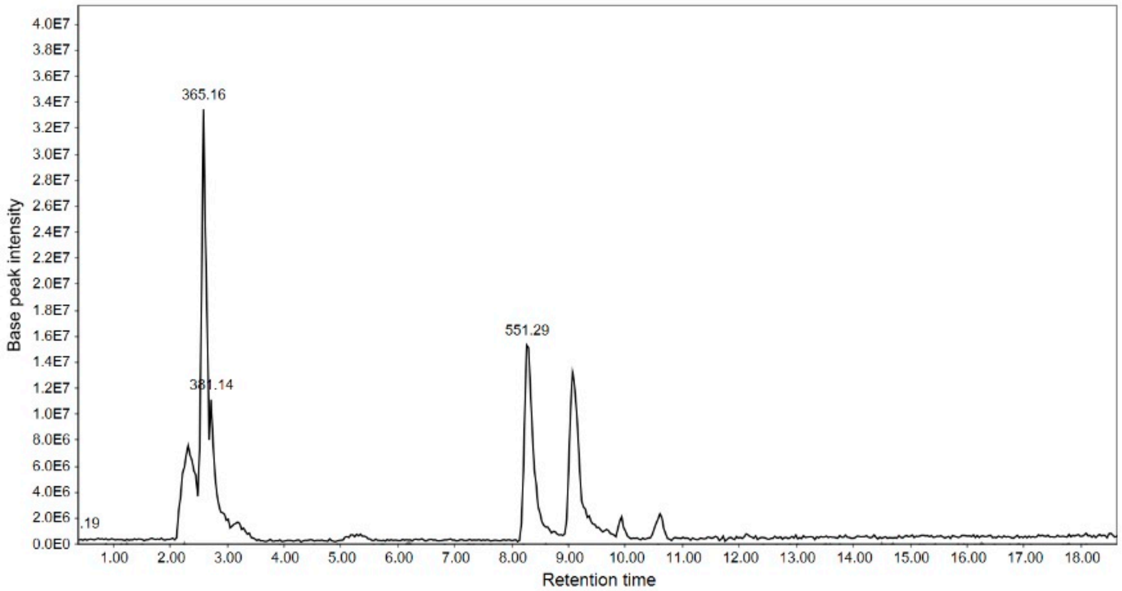

Figure S2: LC-MS spectra of commercial betanin showing sodium (Mw 22.9 g.mol^{-1}) and sucrose (Mw 342 g·mol^{-1}) adduct through the molecular ion m/z 365 at approximately 2.5 minutes.

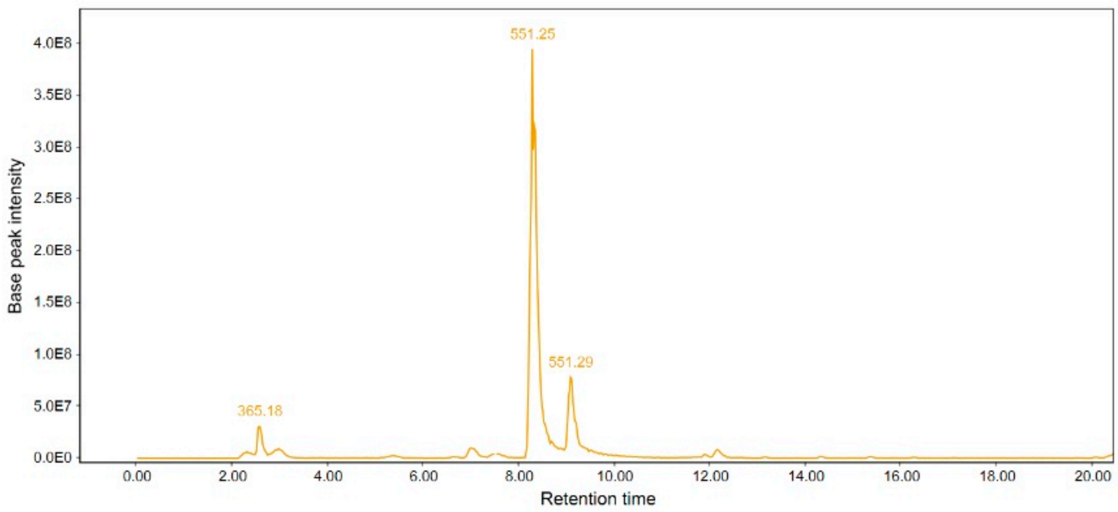

Figure S3: LC-MS spectra of purified betanin showing sodium (Mw 22.9 g·mol^{-1}) and sucrose (Mw 342 g·mol^{-1}) adduct through the molecular ion m/z 365 at approximately 2.5 minutes.

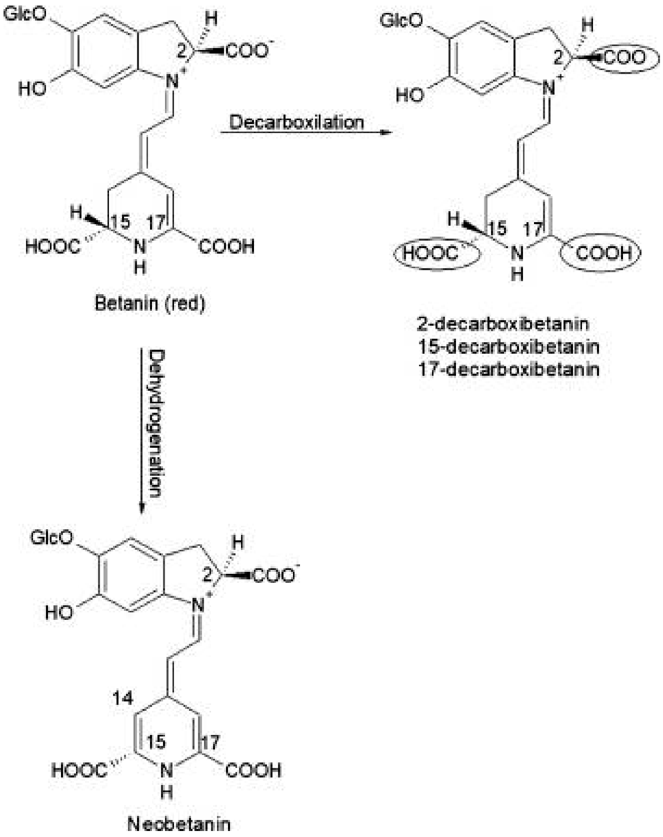

Figure S4: Main degradation products of betanin generated after decarboxylation and/or dehydrogenation generated during processing. Reproduced from Aztatzi-Rugerio et al. [26].

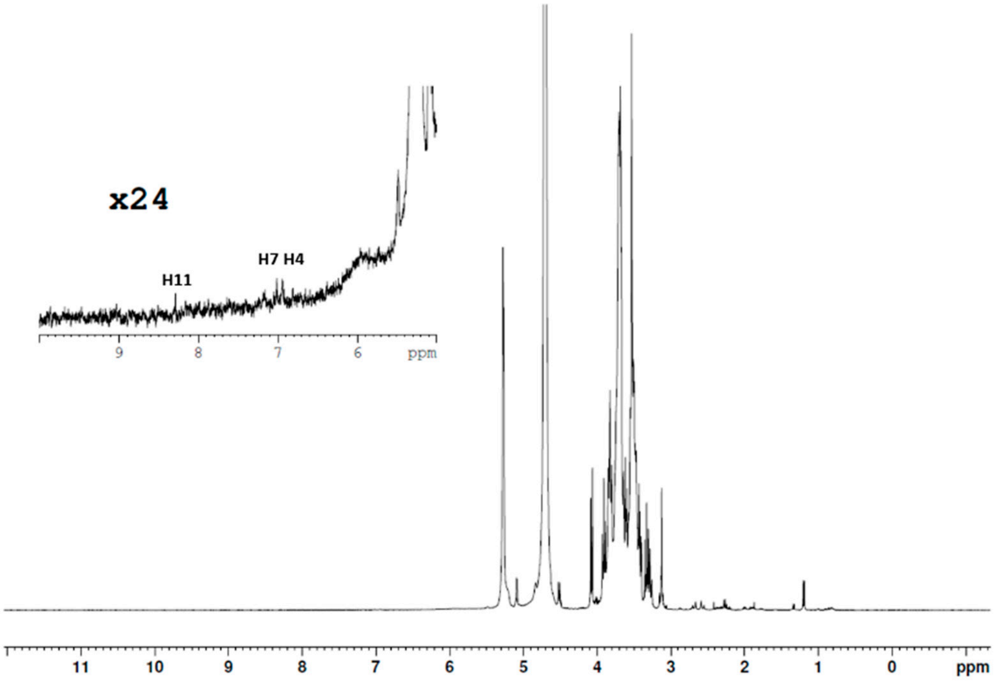

Figure S5: ^{1}H NMR spectrum of commercial betanin at a 24x magnification depicting suppressed H4, H7, and H11 hydrogen signals from betanin molecule due to the high sugar contents.
